# Role of Ubiquitin-conjugating enzyme E2 (UBE2) in two immune-mediated inflammatory skin diseases: a mendelian randomization analysis

**DOI:** 10.1007/s00403-024-02976-4

**Published:** 2024-05-25

**Authors:** Xiaoxue Wang, Zexin Zhu

**Affiliations:** 1 Department of Dermatology, The Second Affiliated Hospital of Xi’an Jiaotong University, Xi’an, China; 2 Department of Surgical Oncology, The Comprehensive Breast Care Center, The Second Affiliated Hospital of Xi’an Jiaotong University, Xi’an, China

**Keywords:** Ubiquitin-conjugating enzyme E2, Psoriasis vulgaris, Atopic dermatitis, Mendelian randomization, Causal inference

## Abstract

Psoriasis vulgaris (PV) and Atopic dermatitis (AD) are the two major types of immune-mediated inflammatory skin disease (IMISD). Limited studies reported the association between Ubiquitin-conjugating enzyme E2 (UBE2) and IMISD. We employed a two-sample Mendelian randomization (MR) study to assess the causality between UBE2 and PV & AD. UBE2 association genome-wide association study (GWAS) data were collected. The inverse variance weighted (IVW) method was utilized as the principal method in our Mendelian randomization (MR) study, with additional using the MR-Egger, weighted median, simple mode, and weighted mode methods. The MR-Egger intercept test, Cochran’s Q test, MR-Pleiotropy RESidual Sum and Outlier (MR-PRESSO) and leave-one-out analysis were conducted to identify heterogeneity and pleiotropy, colocalization analysis was also performed. The results showed that Ubiquitin-conjugating enzyme E2 variant 1 (UBE2V1) was causally associated with PV (OR = 0.909, 95% CI: 0.830–0.996, *P* = 0.040), Ubiquitin-conjugating enzyme E2 L3 (UBE2L3) was causally associated with AD (OR = 0.799, 95% CI: 0.709–0.990, *P* < 0.001). Both UBE2V1 and UBE2L3 may play protective roles in patients with PV or AD, respectively. No other significant result has been investigated. No heterogeneity or pleiotropy was observed. This study provided new evidence of the relationship between UBE2V1 and PV, UBE2L3 and AD. Our MR suggested that UBE2V1 plays an inhibitory role in PV progression, UBE2L3 plays an inhibitory role in AD. These could be novel and effective ways to treat PV and AD.

**Supplementary Information:**

The online version contains supplementary material available at 10.1007/s00403-024-02976-4.

## Introduction

Psoriasis vulgaris (PV) and Atopic dermatitis (AD) are the two major types of immune-mediated inflammatory skin disease. The pathogenesis of AD and PV are also complex. Psoriasis has a relatively high incidence and prevalence worldwide, accordingly, PV is affecting 2–3% of adults in Europe and the USA, and 125 million people globally [1–3]. Likewise, AD affects up to 20% of the world population [4]. Both of them are characterized by pruritus and xerosis [3, 5]. These skin manifestations severely affect the quality of life because of these skin manifestations. A specific aspect of these two diseases progression involves substantial psychological disability, with up to 20% of those affected reporting symptoms of depression [3, 6]. Treatment options for them are similarity in general, which include topical therapies, phototherapy, oral medications, and biologic agents [7, 8]. Patients have severe disease, which cannot be controlled with the exclusive use of topical therapies (e.g., corticosteroids). More severe disease controlled with topical therapies with systemic biologic agents such as adalimumab, ustekinumab, secukinumab or ixekizumab (for PV), dupilumab (for AD) [2, 4]. Recent years, developments focused on new biologic agents got some achieve results, however, given the highly heterogeneous character of these diseases, biologic therapies are used by only a minority of patients [2, 8].

Small ubiquitin-like modifier (SUMO), is a post-translational modification of proteins [9]. SUMO takes place via a cascade reaction of the dimeric SUMO activators E1, E2 and a limited set of E3 ligases [9, 10]. The only E2 enzyme known for SUMO modification is Ubiquitin-conjugating enzyme E2 (UBE2). UBE2 family members are key to SUMOylation and contributes to the SUMO linkage to the substrate [9, 10], play important roles in numerous physiological and pathological processes. For instance, Ubiquitin-conjugating enzyme E2 C (UBE2C) participated in the progression of thyroid carcinoma (THCA), may play the dual role of both oncogene and tumor suppressor gene [11]. High expression level of Ubiquitin-conjugating enzyme E2 H (UBE2H), was identified in the metastatic malignant pleural tumor, regulated the epithelial-mesenchymal transition (EMT) program and metastasis in lung adenocarcinoma (LUAD) [12]. Ubiquitin-conjugating enzyme E2 L3 (UBE2L3) is associated with increased susceptibility to chronic Hepatitis B virus (HBV) infection in adults [13]. Overexpression of UBE2S was associated with poor overall survival and disease-free survival in patients with liver cancer [14].

Emerging studies also reported the relationship between UBE2 and skin disease. Ubiquitin-conjugating enzyme E2 T (UBE2T) mRNA was significantly highly expressed in malignant melanoma tissues, associated with the poor overall survival rate of malignant melanoma patients [15]. Ubiquitin-conjugating enzyme E2 O (UBE2O) decreases the level of SMAD family member 6 (SMAD6), activates bone morphogenetic protein 2 (BMP2), enhance wound healing through promotion of angiogenesis [16]. Ubiquitin-conjugating enzyme E2 C (UBE2C) is a hub gene might have a role in development and progression of the psoriasis [17]. Ubiquitin-conjugating enzyme E2 N (UBE2N) educe TRAF6 activity and represents a promising novel strategy for targeting autoimmune and chronic inflammatory diseases [18]. Meanwhile, studies focusing on the functions of UBE2 in PV and AD are still limited. The causal relationship between UBE2 and PV&AD remains unclear.

Mendelian randomization (MR) utilizes one or more genetic variants as instrumental variables (IVs) based on genome-wide association studies (GWAS). MR studies can infer the causal effects of exposure on an outcome. To our knowledge, no study has yet investigated the causal effect of UBE2 on PV&AD risk using Mendelian randomization. Our investigation aimed to explore the UBE2 risk variants as instrumental variables for PV and AD utilizing two-sample MR.

## Methods

### Study design

**Fig. 1:**
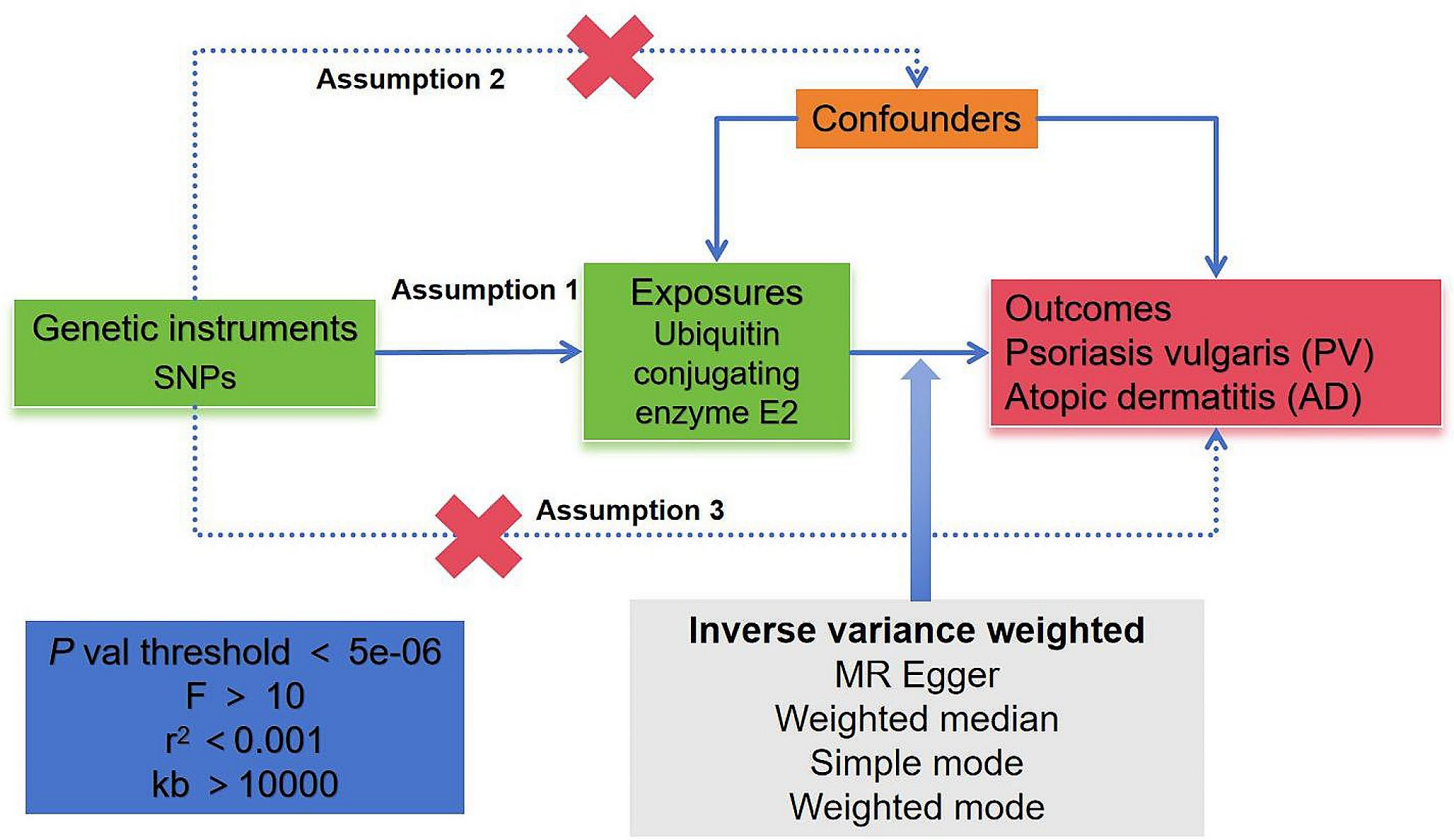
Flowchart schematic diagram followed by the MR analysis’ principal of this study

According to the MR framework (Fig. 1), three key assumptions are included: (1) Relevance Assumption: This initial assumption involves using SNPs significantly associated with exposures (UBE2) as IVs. (2) Independence Assumption: The second assumption stipulates that these SNPs (IVs) should remain uncorrelated with the relevant confounding factor, which are factors linked to both the exposure and the corresponding outcome (PV and AD). (3) Exclusivity Assumption: Lastly, the third assumption dictates that the SNPs (IVs) should exclusively influence outcome susceptibility through their direct impact on the exposure, without any other significant associations with the outcome (PV and AD) itself [19, 20].

### Instrumental variables (IVs) selection

We utilized summary data from the INTERVAL study accessed at https://gwas.mrcieu.ac.uk. Different subtypes of UBE2, including UBE2B, UBE2D4, UBE2G2, UBE2J1, UBE2J2, UBE2L3, UBE2N, UBE2T, and UBE2V1, were identified, summary data were accessed [21].

UBE2-related IVs for MR analysis followed specific criteria: SNPs were not closely related (at least 10,000 kpb apart) and exhibited a low level of linkage disequilibrium (r ^2^ ≤ 0.001), threshold (*P* < 5e-06) SNPs reaching genome-wide significance, and the consistently criteria for reverse MR studies of positive results.

### Genetic association of SNPs with outcomes risk

Summary statistics for PV and AD risk, were obtained from the IEU open GWAS database https://gwas.mrcieu.ac.uk. Accession numbers ebi-a-GCST90018907 (PV, 5,072 cases and 478,102 controls) and ebi-a-GCST90018784 (AD, 6,224 cases and 475,075 controls) [22], summary data was accessed via the TwoSampleMR programme in R. All participants provided informed written consent, and all studies were reviewed and approved by institutional ethics review committees at the involved institutions.

### MR analysis

MR analysis was performed between UBE2 and PA&AD. For significance results, they were treated alternately as exposure and outcome to disentangle reverse causality (mentioned in Fig. 1). In the exposure-outcome analysis, we employed MR with more than two SNPs serving as IVs. Various MR methodologies were applied to infer causal relationships for a total of nine types of UBE2 and two skin diseases. Generally, MR methods included inverse variance weighted (IVW), MR-Egger, MR-Egger with a simulation extrapolation (SIMEX), weighted median, simple mode, and weighted mode methods. IVW is known as the most commonly used statistical approach in MR analysis, it was chosen as the primary method for evaluating causal effects [19, 20, 23].

### Sensitivity analysis

Cochrane’s Q test was assessed the heterogeneity of the selected SNPs, *P*> 0.05 indicated the lack of heterogeneity. When significant heterogeneity was detected, the random effects model was applied. The MR-Egger intercept test was used to evaluate the presence of potential horizontal pleiotropy influencing the MR results. Scatter plot was employed to visualize the causal-effect estimates for individual variants, showing the SNP-outcome associations in relation to SNP-exposure associations. Thereafter, we performed a “leave-one-out” analysis to examine the stability of the results in the context of a single SNP’s influence and presented the findings in a forest plot [19, 20, 23].

### Colocalization analysis

We performed a colocalization analysis to investigate the relationship between MR findings that were statistically significant in outcomes. The analysis was conducted using the “coloc” package. Within the eQTL dataset, we established the prior likelihood for cis-eQTL. Five mutually exclusive hypotheses about causal variance sharing between two traits: (H0–H4). We deemed colocalization to be significant if the posterior probability (PPH4) was greater than 0.70 [24, 25].

### Protein-protein interaction (PPI) network

PPI network is an organization of interacting proteins produced by biochemical events that serve a specific biological function as a complex. The PPI network has been used in various biological analyses [26, 27]. Hence, we conducted a PPI network based on UBE2. PPI network was conducted by the STRING database (https://string-db.org/), visualized by Cytoscape 3.10.1.

### Statistical analysis

All statistical analyses were conducted in R software (V.4.3.2) using the TwoSampleMR package (V.0.5.8). The statistical significance level is *P* <0.05. Pooled ORs with 95%CI were calculated. Bonferroni-correction has been used, *P*-value above the Bonferroni-corrected threshold, but lower than 0.05 considered suggestive evidence for a potential causal association [28, 29].

## Results

### Instrumental variables selection

Based on established quality control criteria, SNPs associated with UBE2 were selected as IVs. Table S1 showed the essential information of different subtype of UBE2. The F-statistics of these SNPs exceeded the threshold of 10, signifying their robust representation of UBE2 in the MR analysis. The SNPs included in the exposure data are detailed in Supplementary Table 5.

### MR analysis

**Fig. 2:**
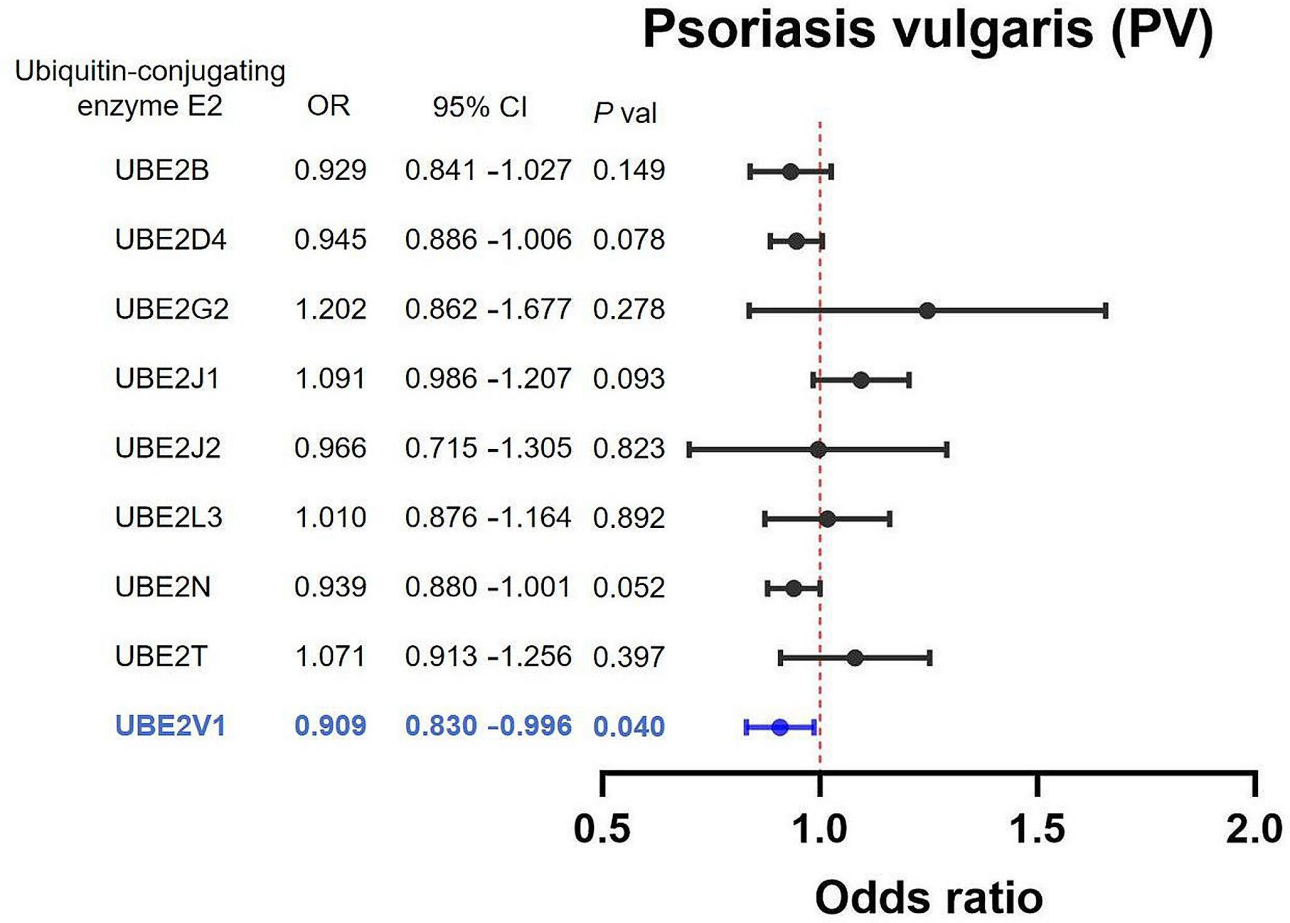
Forest plot of Mendelian randomization analysis for Ubiquitin-conjugating enzyme E2 (UBE2) and Psoriasis vulgaris (PV) risk. The inverse-variance weighted method to investigate the causal associations between UBE2 (UBE2B, UBE2D4, UBE2G2, UBE2J1, UBE2J2, UBE2L3, UBE2N, UBE2T, UBE2V1) and PV. OR, odds ratio; CI, confidence interval; Blue: statistically significant results of UBE2V1 on PV, *p* < 0.05

**Fig. 3:**
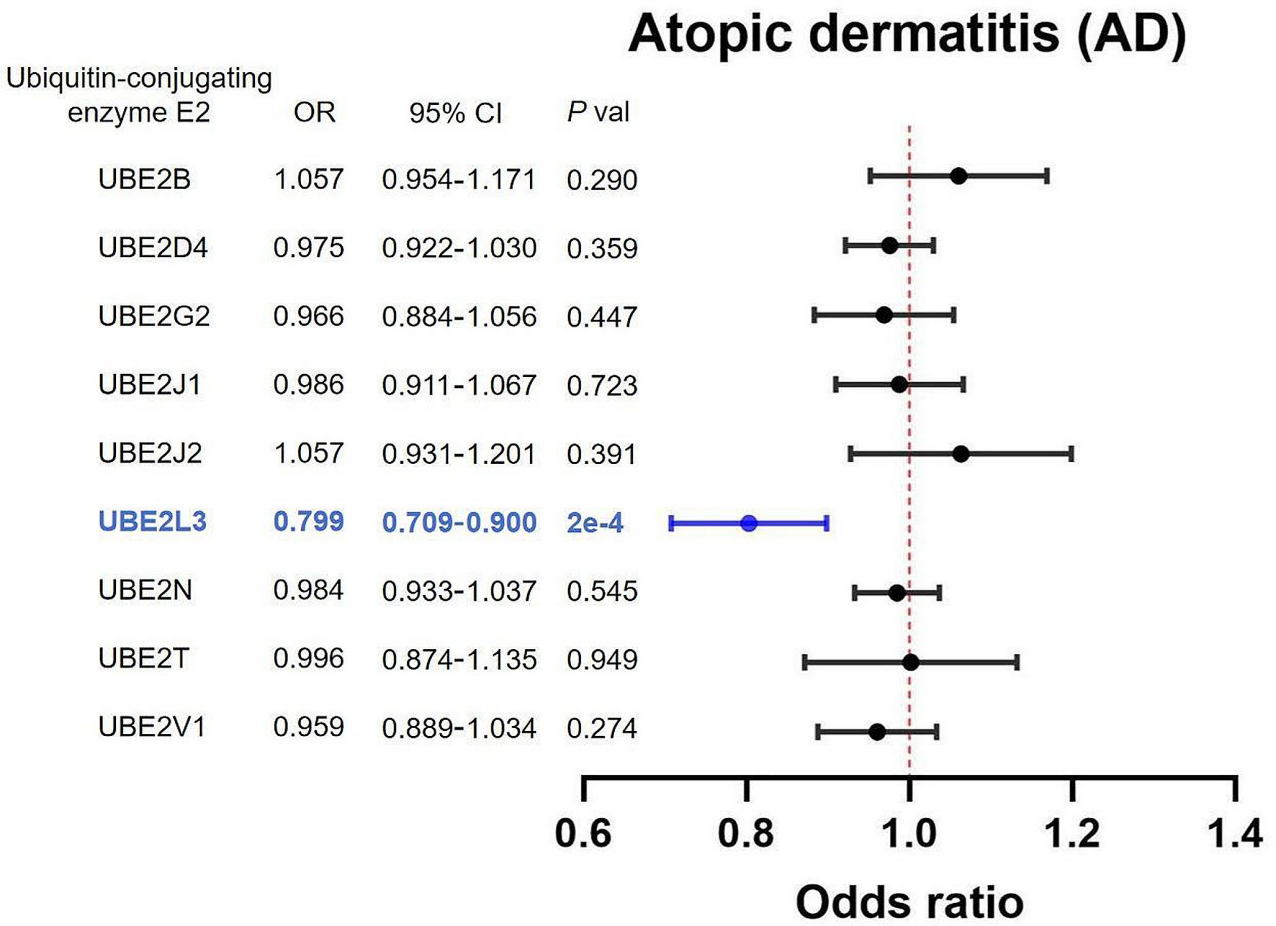
Forest plot of Mendelian randomization analysis for Ubiquitin-conjugating enzyme E2 and and Atopic dermatitis (AD) risk. The inverse-variance weighted method to investigate the causal associations between UBE2 (UBE2B, UBE2D4, UBE2G2, UBE2J1, UBE2J2, UBE2L3, UBE2N, UBE2T, UBE2V1) and AD. OR, odds ratio; CI, confidence interval; Blue: statistically significant results of UBE2L3 on AD, *p* < 0.05

**Fig. 4:**
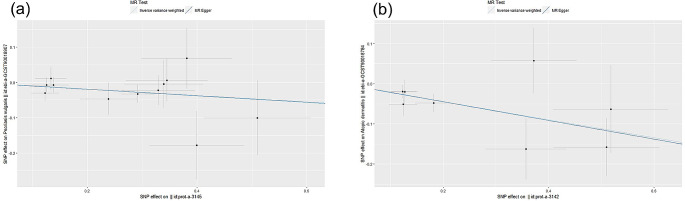
Scatter plots showing significant causal effects between (**a**) UBE2V1 and PV, (**b**) UBE2L3 and AD showed a significant negative causal effects

A total of 9 causal UBE2 were tested, after which we conducted the two sample MR analysis between causal UBE2 and two skin diseases (Table S2). The IVW MR analysis demonstrated that Ubiquitin-conjugating enzyme E2 variant 1 (UBE2V1) was associated with a decreased risk of AD (OR = 0.909, 95% CI: 0.830–0.996, *P* = 0.040, detailed in Table S3), similarity, Ubiquitin-conjugating enzyme E2 L3 (UBE2L3) was associated with a decreased risk of PV (OR = 0.799, 95% CI: 0.709–0.990, *P* < 0.001, detailed in Table S4), no other significant association between UBE2 and PV&AD was observed (Fig. 2 for PV, Fig. 3 for AD). Using the MR-Egger, the relationships of UBE2V1-PV and UBE2L3-AD had the same protective direction, respectively (Fig. 4a, b).

### Sensitivity analysis

According to the Cochran Q test, our IVW-MR analysis results demonstrated no evidence of heterogeneity among the reported results. Furthermore, both the MR-Egger regression and MR-PRESSO analysis results provided evidence that there was no significant horizontal pleiotropy (Table 1). The symmetric funnel plot (Fig. S1) indicated no evidence of horizontal pleiotropy. We also conducted leave-one-out method to identify and delete abnormal instrumental variables. The results showed the robustness of our results (Fig. S2). These results suggest that the MR analysis results were relatively stable.

**Table 1:** Sensitivity analysis of our MR

Exposure	Outcome	Q	*P*-value for Cochran Q test	Egger-intercept	*P*-value for MR-Egger intercept	*P*-value for MR-PRESSO Global test
UBE2V1	PV	6.217	0.858	-0.001	0.968	0.898
UBE2L3	AD	5.806	0.656	0.001	0.943	0.658

### Reversed MR

We also conducted the reversed MR, we identified SNPs associated with PV or AD were selected as IVs, respectively. After Reversed MR, no significant results were detected in our reversed MR (Table 2), which demonstrated the reliability and the stability of our MR. The SNPs included in the PV and AD are detailed in Supplementary Tables 6 and 7.

**Table 2:** Reverse causality between UBE2V1 and PV, UBE2L3 and AD

Exposure	Outcome	*Methods*	*P*
		Inverse variance weighted	0.088
		MR-Egger	0.509
PV	UBE2V1	Weighted median	0.229
		Simple mode	0.414
		Weighted mode	0.343
		Inverse variance weighted	0.760
		MR-Egger	0.361
AD	UBE2L3	Weighted median	0.572
		Simple mode	0.079
		Weighted mode	0.581

### Colocalization analysis

Two colocalization analyses showed that there was no shared genetic variation region between UBE2V1 and PV, UBE2L3 and AD (Fig. S3), suggesting that the genetic variant SNPs were reducing PV or AD risk through their impact on exposures. Shared regions of genetic variation were not observed.

### Protein-protein interaction (PPI) network based on ubiquitin-conjugating enzyme E2

**Fig. 5:**
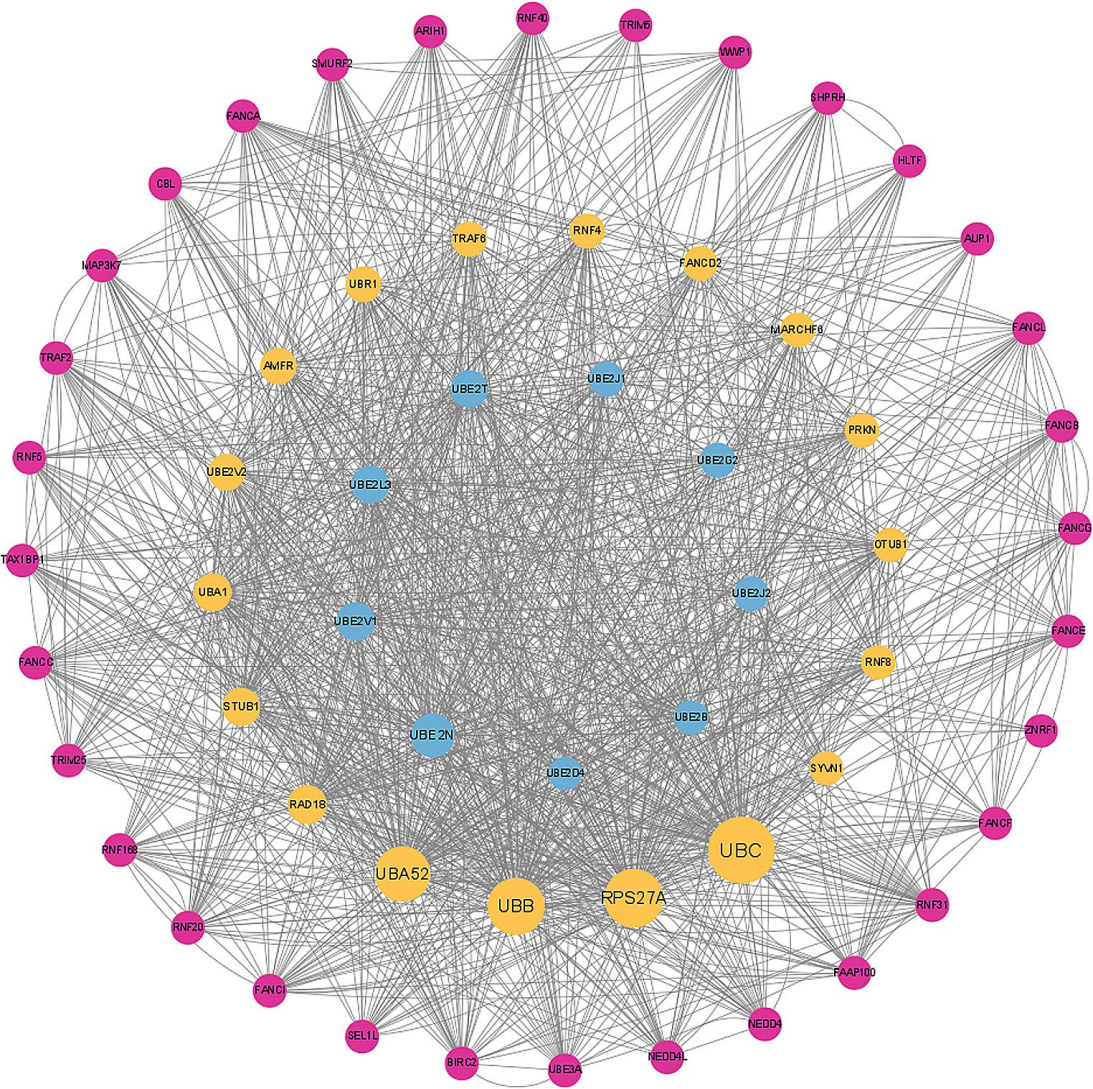
Protein-protein interaction (PPI) network based on Ubiquitin-conjugating enzyme E2

As mentioned, our colocalization analysis showed weak evidence of shared genetic variation in the UBE2V1 with PV, UBE2L3 with AD. This suggests that, UBE2V1 &UBE2L3 take part in the progression of PV&AD with other factors, PPI networks are a powerful tool to study biological processes, reveal protein-protein interactions-both physical interactions as well as functional associations (Fig. 5).

## Discussion

We conducted a MR analysis to investigate the causal relationship between UBE2 and two skin disease (PV & AD) utilizing GWAS summary-level data. Our results showed that UBE2V1 has negative causal relationship on PV, UBE2L3 has negative causal relationship on AD. In addition, sensitivity analyses supported the validity of the results. To the best of our knowledge, the current analysis of UBE2 on PV or AD is limited, the relationship between UBE2 and skin disease has rarely been reported.

Studies reported that UBE2L3 play a role in different tumors. For example, UBE2L3 was generally upregulated in clinical liver cancer samples compared to non-tumour samples, may be an important pro-tumorigenic factor in liver carcinogenesis [30]. In addition, UBE2L3 promotes lung adenocarcinoma invasion and metastasis [31]. On the other hand, UBE2L3 promotes squamous cell carcinoma progression [32]. On the contrast, overexpression of UBE2L3 decreases E7 protein levels and suppresses head and neck tumor growth in vivo [33]. Intriguingly, studies also reported UBE2L3 plays a role in immune-related disease, UBE2L3 influence autoimmunity in patients with Systemic lupus erythematosus (SLE) [34]. More importantly, UBE2L3 acted as a Hub gene, demonstrated potential predictive ability for efficacy in AD treatment [35], UBE2L3 Reduces E3 Ubiquitin-protein ligase TRIM21 expression and IL-1β secretion in epidermal keratinocytes and improves psoriasis-like skin, indicated that UBE2L3 may be a protective biomarker in the epidermis of psoriasis [36]. Meanwhile, our MR did not find the causal relationship between UBE2L3 and PV.

UBE2V1 plays an important role in protein aggregate formation, study indicated that inhibition of UBE2V1 may provide a novel therapeutic target to treat cardiac proteinopathies [37]. In addition, UBE2V1 of aqueous humor expressed in the patients with Retinoblastoma (RB) [38], similarly, high expression of UBE2V1 was correlated to the poor prognosis of patients with cervical cancer [39]. The role of UBE2V1 in skin disease is unfamiliar, accordingly, UBE2V1 enables cellular responses toward fibroblast growth factor signaling in endothelium, acts as a key modulator for angiogenesis [40]. To our knowledge, there is no study reported the relationship between UBE2V1 and PV or AD.

As mentioned, PV and AD are common chronic inflammatory skin diseases that are mediated by immune cells [41], Studies also reported that UBE2 play a role in immune-inflammation process. Deletion of UBE2L3 in mice leads to excessive mature IL-1β production, could induce neutrophilic inflammation and disease following inflammasome activation [42], likewise, the UBE2M is essential for the maintenance of Regulatory T cell fitness [43]. Our results also indicated that UBE2 take part in these two immune-inflammatory skin diseases.

### Strengths and limitations

Our study utilized MR analysis to explore the causal effect of various UBE2 on PV and AD, implied that UBE2 may be a new target for PV or AD. Finding new biomarkers may provide us a better understanding of the pathogenesis of PV and AD, and enable better evaluation the condition of patients with these two skin diseases. Our results demonstrated that the relationship between UBE2 and PV or AD is worthy attention. The findings of our study could extend to experimental design and clinical practice, further research is needed to elucidate the function of UBE2 on PV and AD.

There are several limitations to our study. First, due to the original GWAS statistics, we were unable to divide the cohorts or perform subgroup analyses. Second, our analysis only included individuals of the European population. Although using a single European population to investigate causal relationships can minimize population stratification bias, it is important to interpret these findings with caution regarding their applicability to other populations.

Our findings resulted UBE2V1 has nominal causal connections with PV, but these correlations vanished after applying the Bonferroni correction (0.040>0.05/9 = 0.0056). It is important to note that the Bonferroni correction can result in false negatives [28, 29].

Colocalization analysis showed weak evidence of shared genetic variation in the UBE2V1 with PV, UBE2L3 with AD. This was because colocalization generally provides more conservative results. Mendelian randomization is distinct from colocalization, as colocalization only considers associations at a single genetic region [25]. Accordingly, most exposures used in Mendelian randomization analysis are clinical biomarkers or phenotypic traits, including mRNA expression and protein levels [25]. As descriptions, subtype of UBE2 is kind of enzymes, MR analysis is more suitable.

We have confirmed a causal relationship between UBE2V1 and PV, UBE2L3 and AD, but the mechanism of how UBE2 works remains unclear, further study is required. It should be noted that although we found no evidence of associations between other subtypes of UBE2 and the risk of PV and AD, this does not mean that other subtypes of UBE2 had no impact on PV and AD.

## Conclusion

In conclusion, our MR study has provided the first-ever evidence that UBE2V1 has a negative causal impact on PV, and UBE2L3 has an negative causal impact on AD. Which indicated the UBE2V1 and UBE2L3 may function as a protect role in PV and AD, provide novel therapeutic target and clinical strategy in the treatment of PV and AD.

### Electronic supplementary material

Below is the link to the electronic supplementary material.

Supplementary Material 1

Supplementary Material 2

Supplementary Material 3

Supplementary Material 4

Supplementary Material 5

## Data Availability

No datasets were generated or analysed during the current study.
