# Supplementary figures and images for: Role of Ubiquitin-conjugating enzyme E2 (UBE2) in two immune-mediated inflammatory skin diseases: a mendelian randomization analysis
Source: Arch Dermatol Res. 2024 May 25;316(6):249. doi: 10.1007/s00403-024-02976-4 (PMC11127807; doi:10.1007/s00403-024-02976-4)

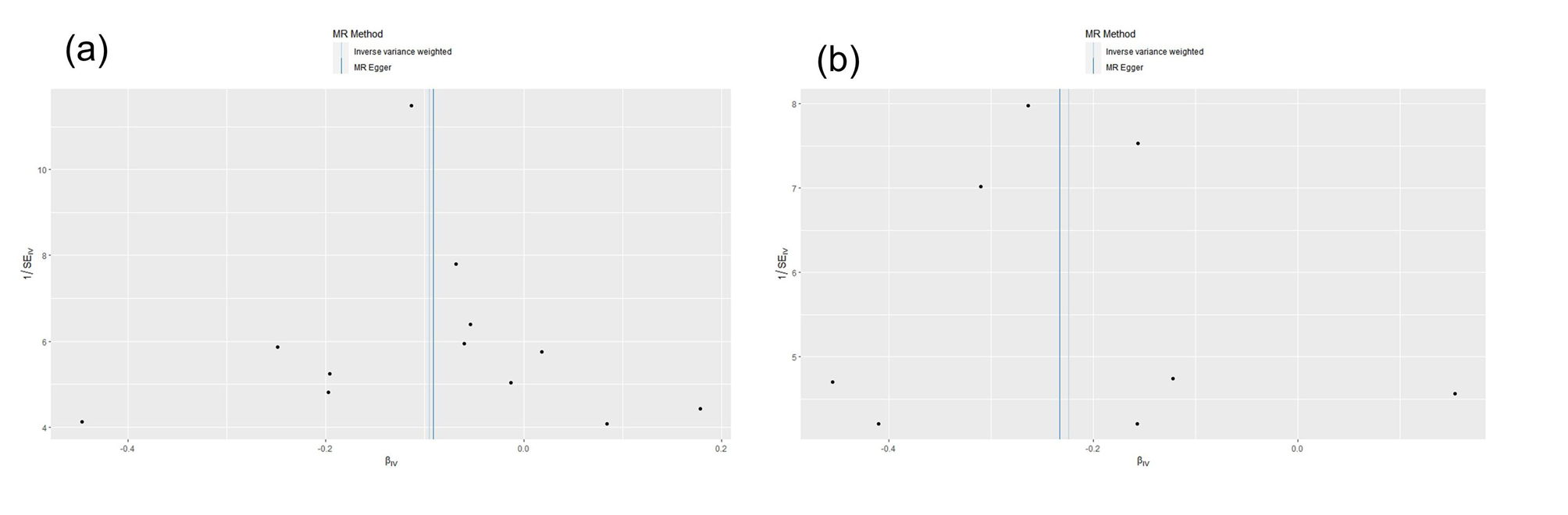

Supplement: Supplementary file 1 — Supplementary Material 1 [file 403_2024_2976_MOESM1_ESM.png]

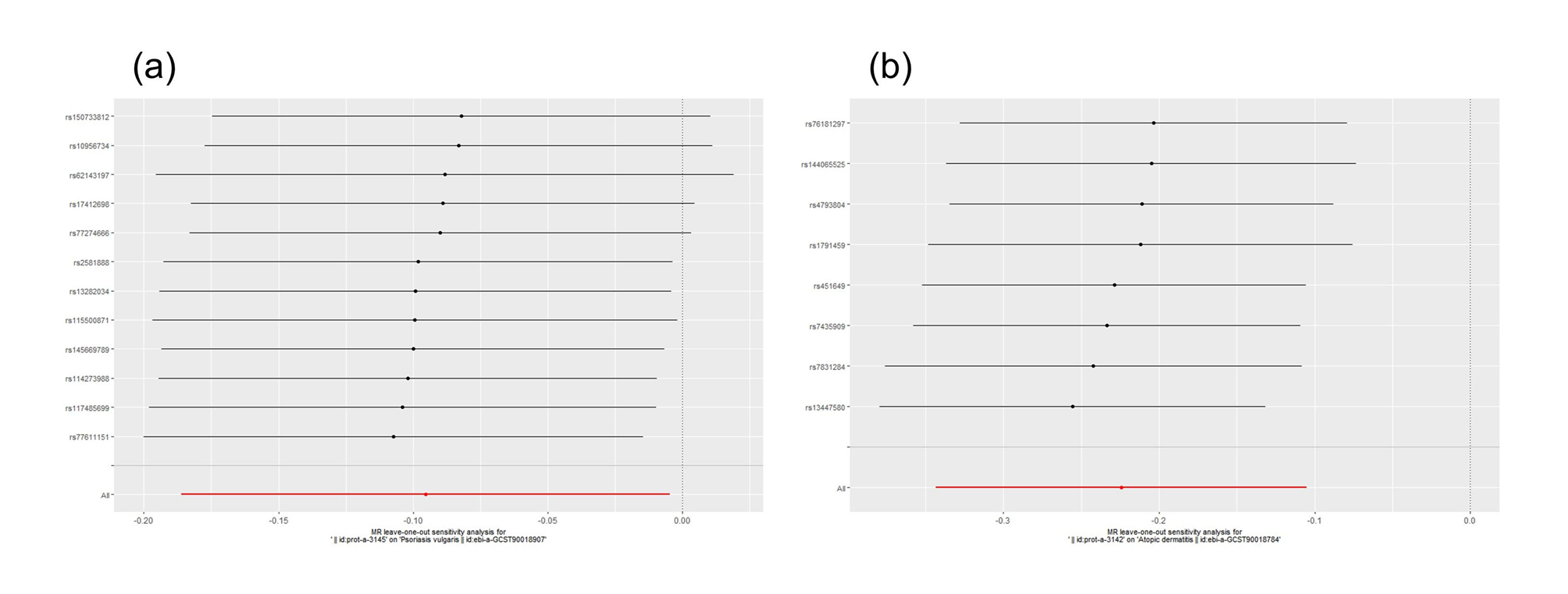

Supplement: Supplementary file 2 — Supplementary Material 2 [file 403_2024_2976_MOESM2_ESM.png]

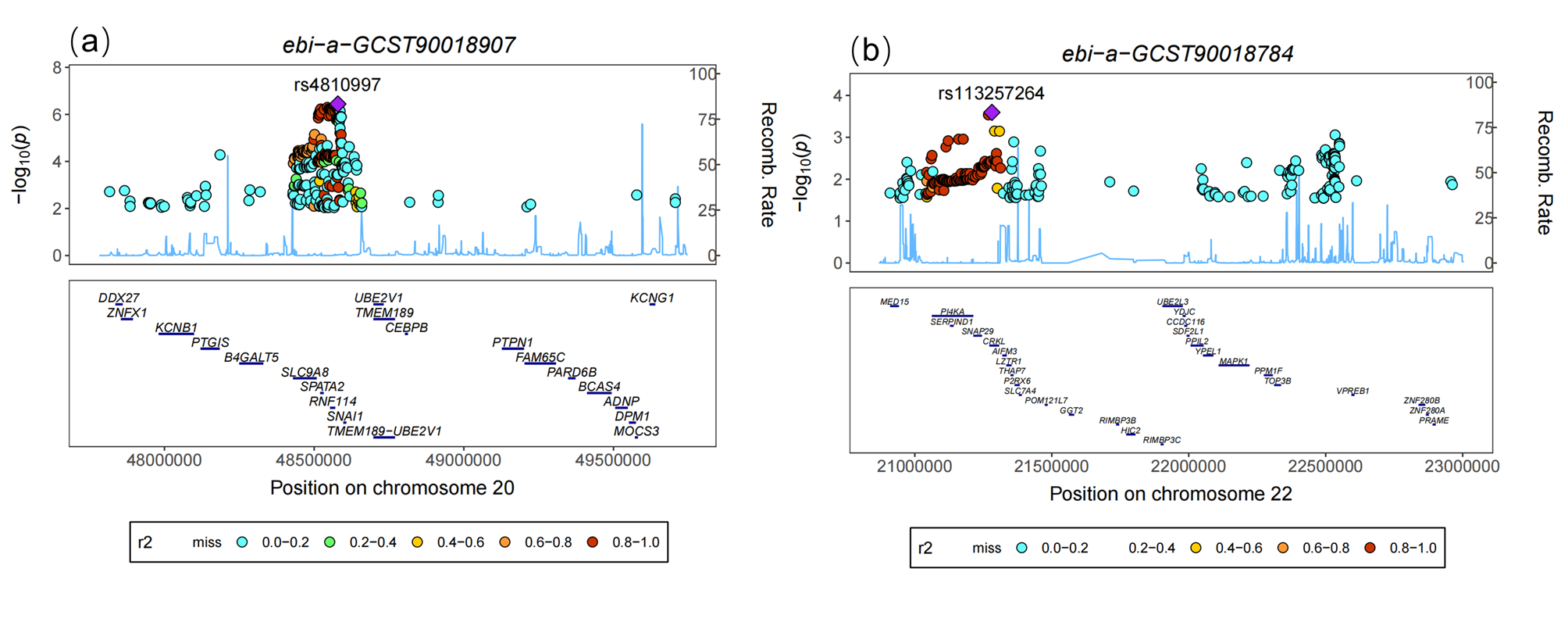

Supplement: Supplementary file 3 — Supplementary Material 3 [file 403_2024_2976_MOESM3_ESM.png]
