# Supplementary material for: Role of Ubiquitin-conjugating enzyme E2 (UBE2) in two immune-mediated inflammatory skin diseases: a mendelian randomization analysis
Source: Arch Dermatol Res. 2024 May 25;316(6):249. doi: 10.1007/s00403-024-02976-4 (PMC11127807; doi:10.1007/s00403-024-02976-4)
Supplement: Supplementary file 4 — Supplementary Material 4 [file 403_2024_2976_MOESM4_ESM.doc]

**Table S1: Information on exposure data included in the study**

| Ubiquitin-conjugating enzyme E2 | IEU GWAS ID | Instrumental variables SNPs |
| --- | --- | --- |
| UBE2B | prot-a-3135 | 15 |
| UBE2D4 | prot-a-3136 | 18 |
| UBE2G2 | prot-a-3137 | 17 |
| UBE2J1 | prot-a-3139 | 11 |
| UBE2J2 | prot-a-3141 | 12 |
| UBE2L3 | prot-a-3142 | 9 |
| UBE2N | prot-a-3143 | 19 |
| UBE2T | prot-a-3144 | 7 |
| UBE2V1 | prot-a-3145 | 12 |

**Table S2: Information on outcomes data included in the study**

| Skin disease | IEU GWAS ID | Instrumental variables SNPs (for reverse MR) |
| --- | --- | --- |
| Psoriasis vulgaris (PV) | ebi-a-GCST90018907 | 15 |
| Atopic dermatitis (AD) | ebi-a-GCST90018784 | 24 |

**Table S3: Causal association of UBE2V1 on PV**

| Methods | OR | 95% CI | *P* |
| --- | --- | --- | --- |
| **Inverse variance weighted** | **0.909** | **0.830-0.996** | **0.040** |
| MR-Egger | 0.913 | 0.739-1.127 | 0.416 |
| Weighted median | 0.922 | 0.815-1.042 | 0.192 |
| Simple mode | 0.939 | 0.788-1.118 | 0.494 |
| Weighted mode | 0.918 | 0.796-1.058 | 0.263 |
| MR-Egger (SIMEX) | 1.001 | 0.964-1.039 | 0.080 |

**Table S4: Causal association of UBE2L3 on AD**

| Methods | OR | 95% CI | *P* |
| --- | --- | --- | --- |
| **Inverse variance weighted** | **0.799** | **0.709-0.900** | **2.3e-4** |
| MR-Egger | 0.792 | 0.610-1.029 | 0.131 |
| Weighted median | 0.812 | 0.691-0.955 | 0.012 |
| Simple mode | 0.833 | 0.662-1.049 | 0.164 |
| Weighted mode | 0.802 | 0.648-0.991 | 0.080 |
| MR-Egger (SIMEX) | 1.007 | 0.962-1.052 | 0.304 |
